# Multiomics analysis provides new insights into the regulatory mechanism of carotenoid biosynthesis in yellow peach peel

**DOI:** 10.1186/s43897-023-00070-3

**Published:** 2023-11-03

**Authors:** Jiarui Zheng, Xiaoyan Yang, Jiabao Ye, Dongxue Su, Lina Wang, Yongling Liao, Weiwei Zhang, Qijian Wang, Qiangwen Chen, Feng Xu

**Affiliations:** 1 College of Horticulture and Gardening, Yangtze University, Jingzhou, 434025 China; 2 School of Biology and Agriculture, Shaoguan University, Shaoguan, 512005 China; 3 Guangdong Provincial Key Laboratory of Utilization and Conservation of Food and Medicinal Resources in Northern Region, Shaoguan University, Shaoguan, 512005 China

**Keywords:** Yellow peach peel, Carotenoid, miRNA, MYB, Degradome

## Abstract

Carotenoids, as natural tetraterpenes, play a pivotal role in the yellow coloration of peaches and contribute to human dietary health. Despite a relatively clear understanding of the carotenoid biosynthesis pathway, the regulatory mechanism of miRNAs involved in carotenoid synthesis in yellow peaches remain poorly elucidated. This study investigated a total of 14 carotenoids and 40 xanthophyll lipids, including six differentially accumulated carotenoids: violaxanthin, neoxanthin, lutein, zeaxanthin, cryptoxanthin, and (E/Z)-phytoene. An integrated analysis of RNA-seq, miRNA-seq and degradome sequencing revealed that miRNAs could modulate structural genes such as *PSY2*, *CRTISO*, *ZDS1*, *CHYB*, *VDE*, *ZEP*, *NCED1*, *NCED3* and the transcription factors NAC, ARF, WRKY, MYB, and bZIP, thereby participating in carotenoid biosynthesis and metabolism. The authenticity of miRNAs and target gene was corroborated through quantitative real-time PCR. Moreover, through weighted gene coexpression network analysis and a phylogenetic evolutionary study, coexpressed genes and MYB transcription factors potentially implicated in carotenoid synthesis were identified. The results of transient expression experiments indicated that mdm-miR858 inhibited the expression of PpMYB9 through targeted cleavage. Building upon these findings, a regulatory network governing miRNA-mediated carotenoid synthesis was proposed. In summary, this study comprehensively identified miRNAs engaged in carotenoid biosynthesis and their putative target genes, thus enhancing the understanding of carotenoid accumulation and regulatory mechanism in yellow peach peel and expanding the gene regulatory network of carotenoid synthesis.

**Graphical Abstract:**

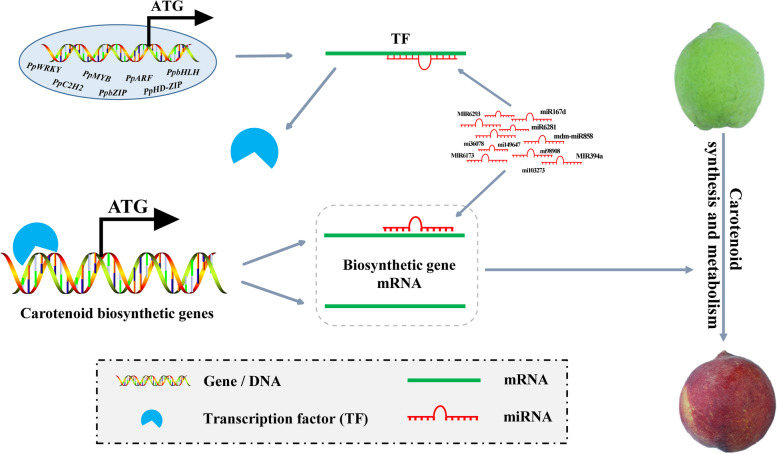

**Supplementary Information:**

The online version contains supplementary material available at 10.1186/s43897-023-00070-3.

## Core

In this study, we conducted a thorough and systematic analysis of carotenoid species and concentrations in yellow peach peel. Through a combined examination of miRNA-seq, RNA-seq, and degradome sequencing, we constructed a plausible regulatory network involving miRNAs and transcription factors that mediate carotenoid synthesis. The validation of miRNA and target gene regulatory components was ascertained.

## Gene & accession numbers

The raw transcriptome and miRNA sequencing data generated from this study have been deposited in the Genome Sequence Archive (GSA) database under accession No. PRJCA017106. AtMYB113 (NP_176811.1), MtWP1 (XP_013442371.1), AtPAP1 (NP_176057.1), CrMYB68 (KY612511.2).

## Introduction

Yellow peach (*Prunus persica* L. cv. Huangjinmi) represents a commercial peach variety esteemed for both its fresh consumption and processing applications. Boasting attributes such as sizable fruit dimensions, elevated yield, and exceptional quality, this cultivar has earned geographical indication recognition across multiple regions. Notably, yellow peach offers a wealth of germplasm resources, a diverse range of flesh hues, and a distinguished reputation for its abundant carotenoid content (Falchi et al. 2013). Carotenoids, a class of 40-carbon isoprene compounds, imbue fruits, flowers, and vegetables with vibrant yellow, orange, and red hues due to the presence of numerous conjugated double bonds (Yuan et al. 2015). Beyond their role in coloration, carotenoids serve as antioxidants, mitigating the risk of chronic ailments, including cancer, cardiovascular disease, and age-related eye disorders. This contributes to the nutritional and health value of fruits and vegetables (Fraser 2004; Moise et al. 2005; Rao and Rao 2007; Falchi et al. 2013; Fiedor and Burda 2014; Mares 2016). Given that carotenoids serve as biosynthetic precursors to vitamin A, which holds protective benefits for human vision (Johra et al. 2020; Krinsky and Johnson 2005), yellow peaches stand as a prime source for carotenoid intake (Krinsky and Johnson 2005; Bernstein et al. 2016). The carotenoid content serves as a pivotal criterion for assessing yellow peach quality, and augmenting this content can elevate overall fruit quality. Consequently, there exists an urgent need to enhance carotenoid levels in yellow peaches through cultivation and biotechnology approaches.

The pathways of carotenoid biosynthesis exhibit a degree of conservation and have been elucidated in various plant species (Cao et al. 2017; Han et al. 2020). The synthesis begins with the MEP and MVA pathways, culminating in the production of geranylgeranyl diphosphate, a direct precursor to carotenoid synthesis. Subsequent steps involve lycopene production through a series of desaturation and isomerization reactions facilitated by enzymes such as phytoene desaturase (PDS), *ζ*-carotene desaturase (ZDS), *ζ*-carotene isomerase (Z-ISO), and carotenoid isomerase (CRTISO). The downstream metabolism of lycopene bifurcates into the *β*, *ε*-carotene and *β*, *β*-carotene branches. The former yields *δ*-carotene, *α*-carotene, *α*-cryptoflavin, and lutein in sequence, while the latter yields *γ*-carotene, zeaxanthin, *β*-carotene, antheraxanthin, violaxanthin, and neoxanthin, among others. Notably, violaxanthin and neoxanthin serve as precursor compounds for abscisic acid (ABA) synthesis.

The biosynthesis of carotenoids is under the regulation of transcription factors (TFs) and miRNAs, however, there are fewer studies on resolving miRNA regulation of carotenoid synthesis. Understanding the pathway and regulatory mechanisms of carotenoid synthesis holds immense significance, as it could pave the way for leveraging genetic engineering to effectively enhance carotenoid content in peaches. Presently, the regulatory mechanisms governing carotenoid synthesis have been extensively investigated across a range of plant species, including *Solanum lycopersicum* (Kachanovsky et al. 2012; Ma et al. 2014; Zhu et al. 2014; Endo et al. 2016), *Capsicum annuum* (Rodriguez-Uribe et al. 2012; Liu et al. 2020), *Citrus sinensis* (Butelli et al. 2012), *Vitis vinifera* (Kobayashi et al. 2004; Mathieu et al. 2005), *Camellia sinensis* (Li et al. 2022), orchids (Li et al. 2020a, b), *Mimulus lewisii* (Sagawa et al. 2016), *Actinidia deliciosa* (Ampomah-Dwamena et al. 2019), and *Medicago truncatula* (Meng et al. 2019). Within these studies, TFs such as bHLH, MYB110, MlRCP, MYB, MtWP1, NAC, NAC and the MBW complex have been identified as key players in carotenoid synthesis. As a highly efficient regulatory component, miRNA also assumes a vital role in plant processes.

Carotenoids have garnered significant attention due to their distinct value. However, prevailing research has primarily centered on variations in carotenoid content (Zhao et al. 2022; Yuan et al. 2015). Conversely, fewer studies have comprehensively explored the diversity of carotenoid species and their contents within peach pericarp, as well as the role of miRNA in regulating carotenoid synthesis. In this study, we quantified carotenoid components within peach peels at three distinct developmental stages through targeted metabolomics. We screened differentially expressed genes related to carotenoid synthesis using transcriptome data. Additionally, miRNAs implicated in carotenoid anabolism were identified via miRNA sequencing. The relationships between miRNAs and target genes were validated through quantitative real-time PCR (qRT‒PCR) and degradome analysis. Employing weighted gene coexpression network analysis (WGCNA), we identified coexpressed gene modules and pinpointed key genes and TFs most pertinent to carotenoid synthesis. By amalgamating these findings, we mapped the regulatory network governing carotenoid synthesis in yellow peach peel. The outcomes of this study shed light on the pattern of carotenoid accumulation during the developmental stages of yellow peach peel. Furthermore, this study unveils the regulatory role of miRNAs in carotenoid synthesis and offers novel insights into the broader regulatory network that underpins carotenoid synthesis.

## Results

### Overview of the carotenoid metabolome in yellow peach peel at different developmental stages

**Fig. 1:**
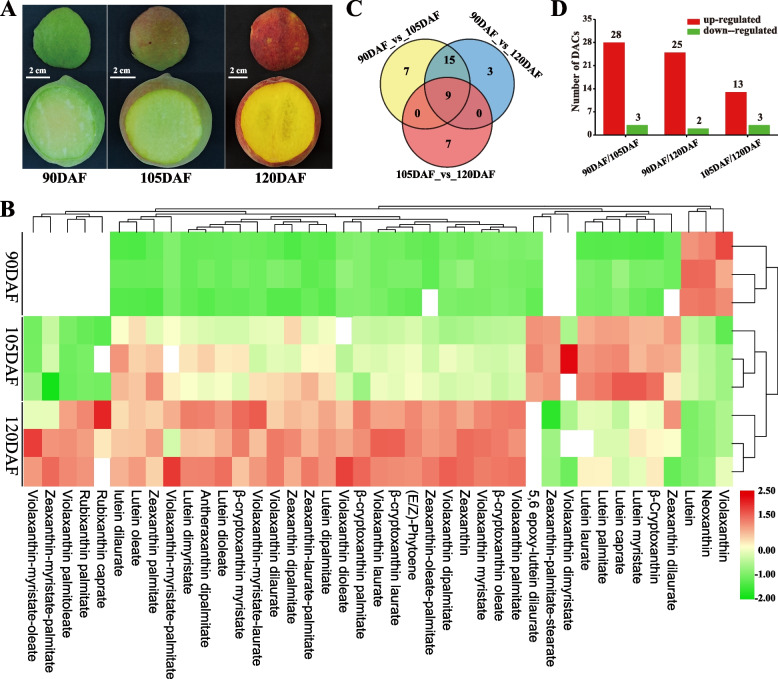
Fruit colors and number of differentially accumulated carotenoid across different developmental stages. **A** Phenotype colors three developmental stages of yellow peach peels. 90DAF, 105DAF, and 120DAF indicate the 90, 105, and 120 days after flowering, respectively. **B** Heatmap depicting the metabolites of DACs. **C**, **D** Quantification of the number of DACs

Metabolomic analysis detected a total of 14 carotenoids and 40 xanthophyll lipids within the yellow peach pericarp across three distinct developmental stages (Table S2). Across the developmental trajectory, the cumulative carotenoid content diminished from 171.46 μg/g DW to 130.53 μg/g DW. Specifically, carotene content exhibited an incremental trend from 90 days after flowering (90DAF) (28.46 μg/g DW) to 120DAF (37.15 μg/g DW), while xanthophyll content experienced a marked decline from 123.55 μg/g DW in 90DAF to 27.53 μg/g DW in 120DAF. Notably, both carotenes and xanthophylls underwent gradual esterification, leading to a decline in their proportion within the overall carotenoid composition, from 88.65% in 90DAF to 49.56% in 120DAF (Table S2). Hierarchical cluster analysis (Fig. 1B; Fig. S1) distinctly classified pericarp carotenoids into three primary clusters aligned with developmental stages. Cluster I exhibited heightened accumulations of neoxanthin, lutein, *α*-carotene, *β*-carotene, violaxanthin, and violaxanthin-dibutyrate in 90DAF. In cluster II, 105DAF displayed elevated accumulations of α-cryptoxanthin, echinenone, *β*-cryptoxanthin, canthaxanthin, and 14 xanthophyll lipids. Cluster III demonstrated elevated levels of zeaxanthin, (E/Z)-phytoene, antheraxanthin, and 27 xanthophyll lipids in 120DAF. Evidently, carotenoid species and contents underwent substantial variations throughout yellow peach peel development. Thus, a subsequent analysis of differentially accumulated carotenoids (DACs) across different developmental stages was performed.

A total of 41 DACs were identified within the peach pericarp across distinct developmental stages (Fig. 1C, D; Table S2), encompassing (E/Z)-phytoene, neoxanthin, lutein, violaxanthin, *β*-cryptoxanthin, zeaxanthin, and 35 xanthophyll lipids. In comparison to 90DAF, 105DAF exhibited upregulated expression of (E/Z)-phytoene and *β*-cryptoxanthin, whereas neoxanthin, lutein, and violaxanthin displayed downregulation. 120DAF demonstrated upregulation of (E/Z)-phytoene, zeaxanthin, and 23 xanthophyll lipids, while lutein and neoxanthin were downregulated. In comparison to 105DAF, 23 xanthophyll lipids were upregulated in 120DAF, while lutein and 2 xanthophyll lipids were downregulated. Notably, lutein and 8 xanthophyll lipids were common DACs. Cluster heatmap analysis (Fig. S1; Table S2) highlighted robust accumulation of violaxanthin (1.83–3.69 μg/g DW), neoxanthin (2.59–13.73 μg/g DW), and lutein (17.1–103.13 μg/g DW) in 90DAF. 105DAF demonstrated elevated levels of *β*-cryptoxanthin (1.44–2.92 μg/g DW), zeaxanthin lipids, violaxanthin lipids, and lutein lipids. The 120DAF stage showed the accumulation of (E/Z)-phytoene, zeaxanthin, violaxanthin lipids, cryptoxanthin lipids, zeaxanthin lipids, lutein lipids, and rubixanthin lipids. Intriguingly, *β*-carotene was maintained at high levels (> 16.8 μg/g DW), while α-carotene and *γ*-carotene remained at low levels (< 0.5 μg/g DW). These findings underscore a distinct pattern of carotenoid accumulation throughout fruit development.

### Analyses of transcriptomic

**Fig. 2:**
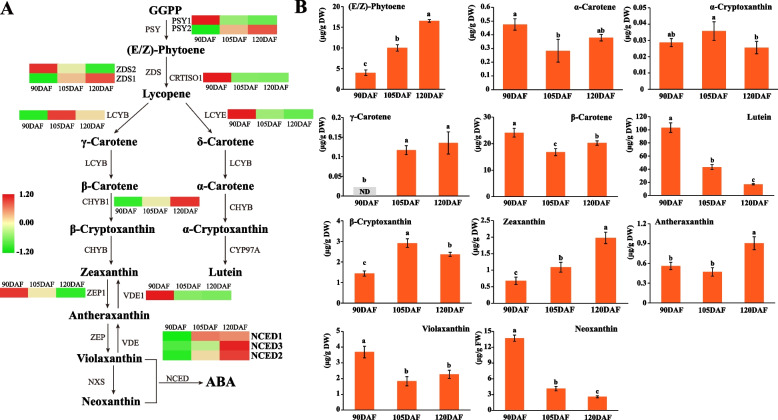
Carotenoid levels and heatmap of carotenoid synthesis genes. **A** Heatmap analysis based on the FPKM values of the genes was performed to illustrate the expression profile of these DEGs in peel of yellow peaches during fruit development. The FPKM value of each gene is the mean of three biological replicates. Red indicates high expression, and green indicates low expression. **B** Carotenoids identified from the peels of yellow peach at different developmental stages. Each bar represents the mean of three biological replicates along with the standard deviation

The expression pattern of carotenoid biosynthesis genes correlates with the pattern of carotenoid accumulation. Consequently, we conducted a comparison of the expression patterns of carotenoid synthesis genes at various developmental stages to unveil the intrinsic mechanisms governing carotenoid accumulation and the shifts in fruit coloration. Across the 90DAF to 120DAF stages, the collective content of total carotenoids and xanthophylls exhibited a decreasing trend, while total carotenes displayed an increasing trend (Table S2). The information presented in Fig. 2B demonstrates that the decline in total lutein content primarily resulted from reduced levels of lutein, neoxanthin, and *β*-cryptoxanthin, while the increase in (E/Z)-phytoene content contributed to the rise in total carotene content. However, phytofluene, *ζ*-carotene, δ-carotene, neurosporene and lycopene were not detected.

RNA-seq analysis unveiled the expression patterns of all genes involved in carotenoid biosynthesis. A total of 14 differentially expressed genes (DEGs) involved in carotenoid biosynthesis were identified (Table S3). Within the carotenoid biosynthesis pathway, PSY is widely recognized as the pivotal rate-limiting enzyme, catalyzing the direct condensation of two geranylgeranyl diphosphate (GGPP) molecules to yield (E/Z)-phytoene (Luo et al. 2013). Notably, *PSY1* exhibited downregulated expression during 90DAF-120DAF, while *PSY2* displayed upregulated expression over the same developmental stages, culminating in the accumulation of (E/Z)-phytoene. The synthesis of lycopene ensued through catalysis by phytoene desaturase, Z-ISO, ZDS, and CRTISO, with the expression of *ZDS* and *CRTISO* genes exhibiting pronounced variations across different developmental stages. *ZDS1* was upregulated throughout 90DAF-120DAF, while *ZDS2* and *CRTISO* were downregulated. The expression level of *LCYE* was downregulated, whereas *LCYB* demonstrated an initial upregulation followed by downregulation during 90DAF-120DAF. *CHYB* exhibited upregulated expression across 90DAF-120DAF. Meanwhile, *ZEP* and *VDE* experienced downregulation throughout 90DAF-120DAF. In addition, *NCED1*, *NCED2*, and *NCED3* were up-regulated with the development of yellow peach peels, especially *NCED3* expression levels were significantly up-regulated at 105DAF and 120DAF stages. By incorporating insights from the KEGG pathway and relevant references, this study mapped the carotenoid synthesis pathway in peach pericarp, encompassing carotenoid content and DEGs (Fig. 2).

### Coexpression network analysis identified genes related to carotenoid synthesis

**Fig. 3:**
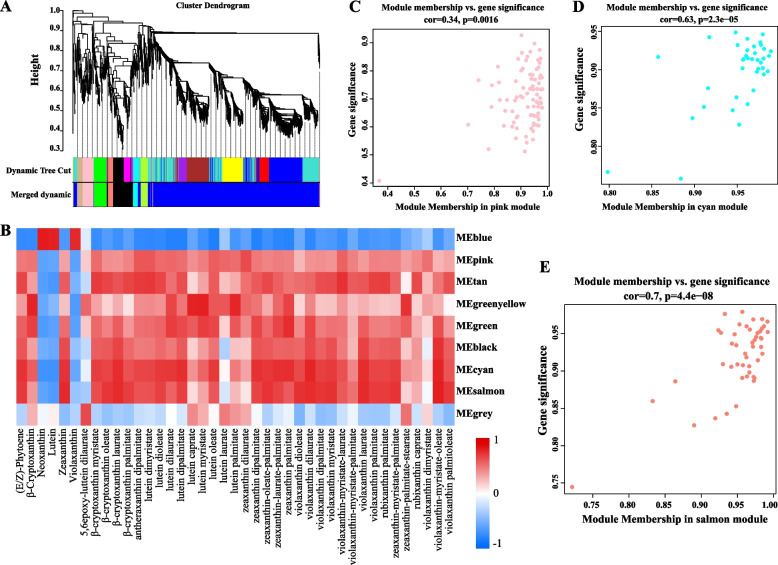
WGCNA of 2958 DEGs (with DACs PCC ≥ 0.90 or ≤  − 0.90). **A** Hierarchical clustering tree (cluster dendrogram) illustrating 9 modules of co-expressed genes identified by WGCNA. Each leaf corresponds to an individual gene, and the major branches define 9 modules, color-coded. **B** Cell values Relationship between modules and carotenoids (detailed information reference Table S5). Rows represent modules, and columns correspond to specific carotenoids. Cell values at intersections depict the correlation coefficient between the module and carotenoid, color-coded based on the scale on the right. Parentheses in cells indicate *P* values. **C-E** Module membership vs. gene significance of pink (**C**), cyan (**D**), salmon (**E**) modules

To delve into the gene regulatory network governing carotenoid synthesis in yellow peach peel, WGCNA was conducted utilizing a nonredundant set of 2,958 DEGs (with carotenoid content Pearson correlation coefficient (PCC) ≥ 0.90 or ≤ -0.90, Table S4). These DEGs were organized into eight major branches, each representing a module (Fig. 3A, B). Modules represent clusters of closely related genes that are coexpressed. The findings revealed a pronounced positive correlation between the salmon and cyan modules and the majority of carotenoids (correlation coefficient > 0.8, *P* < 0.05) (Fig. 3D, E). Moreover, a significant correlation was observed between GS and MM within these modules (Cor > 0.6). This underscores the pivotal role that genes within the salmon and cyan modules play in carotenoid synthesis. Notably, the pink module revealed the presence of *ZDS1*, *NCED2*, and *PSY2*, all of which are implicated in carotenoid synthesis. To further understand the expression trends of genes within the salmon, cyan, and pink modules, heatmaps were generated using the FPKM values of these genes (Table S5; Fig S2A-C). The outcomes highlighted a consistent expression pattern of genes within the pink module during the 105DAF and 120DAF stages, while the genes within the salmon and cyan modules exhibited an increasing trend. These expression patterns suggest vital role of these modules in carotenoid synthesis.

TFs are key factors in plant carotenoid synthesis. Fifteen TFs were identified across the three modules (Table S5). In the salmon module, five TFs were identified, namely, WRKY, NAC, LBD, bHLH, and MYB. Meanwhile, two WRKY TFs were identified within the cyan module. The pink module yielded six TFs, namely, C2H2, ARF, bHLH, bZIP11, HD-ZIP, and NAC. The expression patterns of these transcription factors (Fig. S2D) align closely with the accumulation patterns of carotenoids, suggesting their critical role in carotenoid synthesis.

### Candidate hub genes related to carotenoid synthesis

**Fig. 4:**
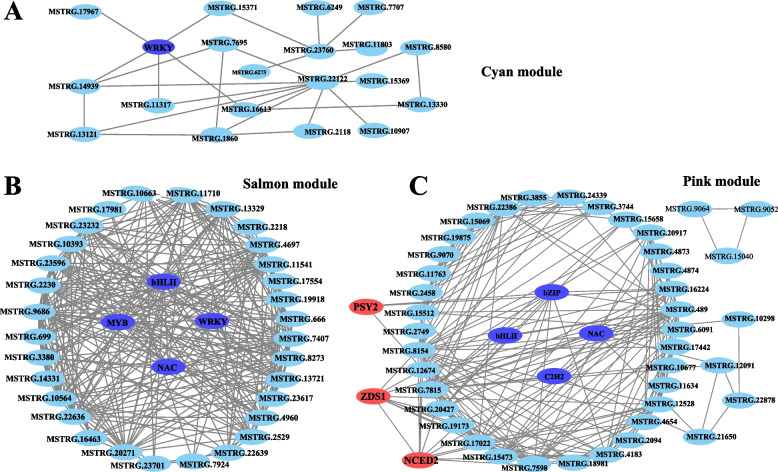
Correlation networks of genes in cyan (**A**), salmon (**B**) and pink (**C**) modules. Displayed are edges with weights surpassing the thresholds 0.3 (**A**), 0.35 (**B**), and 0.35 (**C**). Transcription factors are denoted in blue, while carotenoid synthesis structural genes are shown in red

To further elucidate the interconnections among genes within the modules, we screened for hub genes—genes with highly connectivity—based on the edge weight of the three modules, subsequently constructing a gene network (Fig. 4; Table S6). Within the pink module, a total of 49 genes exhibited robust correlations (with edge weight ≥ 0.35), encompassing 4 TFs, 3 structural genes associated with the carotenoid synthesis pathway, and 2 genes linked to hormone signaling (IAA1 and IAA8). Additionally, notable genes, such as NSP-*INTERACTING KINASE 1* (*NIK1*), *RING-H2 finger protein ATL46* (*ATL46*), *cationic amino acid transporter 6* (*CAT6*), *D-glycerate 3-kinase* (*GLYK*), *4-hydroxy-3-methylbut-2-en-1-yl diphosphate synthase* (*HMDS*), and *FAF-like*, *Stay-green gene* (*SGR*), were identified. A connection was observed between *PSY*2 and bZIP within this module. In the salmon module, 35 genes demonstrated a strong correlation (with edge weight ≥ 0.35). This subset encompassed bHLH, MYB, WRKY, NAC, *proline dehydrogenase 2* (*PRODH2*), the negative regulator of systemic acquired resistance *SNI1*, and the E3 ubiquitin-protein ligase *BOI*. These genes are likely involved in carotenoid synthesis through their potential influence on the expression of gene within the same module. Within the cyan module, 20 genes exhibited a significant correlation (with edge weight ≥ 0.30). Notably, WRKY displayed a pronounced correlation with *MSTRG.14939*. Furthermore, the presence of genes such as a U-box domain-containing protein *17* (*PUB17*), a rust resistance kinase *Lr10-like* gene, a phosphoinositide phospholipase C6 (*PI-PLC6*) gene, and an anthranilate synthase alpha subunit 2 (*ASA2*) gene, all within the chloroplast, underscores their potential roles in the carotenoid synthesis process.

### Identification of MYB transcription factors involved in carotenoid synthesis

To delve into the potential functions of TFs involved in carotenoid synthesis, we identified a total of 106 TFs through correlation analysis (with FPKM > 1, Table S7). This assortment predominantly comprises members of the MYB, ARF, bHLH, bZIP, ERF, HD-ZIP, NAC, and WRKY TF families. Existing studies have underscored the pivotal roles that several members of the MYB family play in carotenoid synthesis (Meng et al. 2019; Li et al. 2022). Hence, this study employed the MYB TFs identified from yellow peach peel to construct a phylogenetic evolutionary tree. This tree was constructed alongside AtMYB113, MtWP1, AtPAP1, and CrMYB68 (Li et al. 2022), all of which have been implicated in carotenoid synthesis. Within this context, PpMYB1 formed a cluster with CrMYB68, while both PpMYB4 and PpMYB9 clustered with MtWP1, AtMYB113, and AtPAP1 (Fig. S3A). Notably, the expression levels of PpMYB4 and PpMYB9 exhibited significant elevation during the 90DAF stage, whereas the expression level of PpMYB1 was notably higher during 105DAF compared to other developmental stages (Fig. S3B). Furthermore, a large number of elements usually bound by MYB, WRKY, NAC, and bZIP TFs were identified in the promoters of these carotenoid synthesis genes (Fig. S4). These observation leads us to speculate that PpMYB1, PpMYB4, and PpMYB9 might serve as crucial candidate regulators in the realm of carotenoid biosynthesis.

### Screening and functional identification of miRNAs involved in carotenoid synthesis in yellow peach peel

**Fig. 5:**
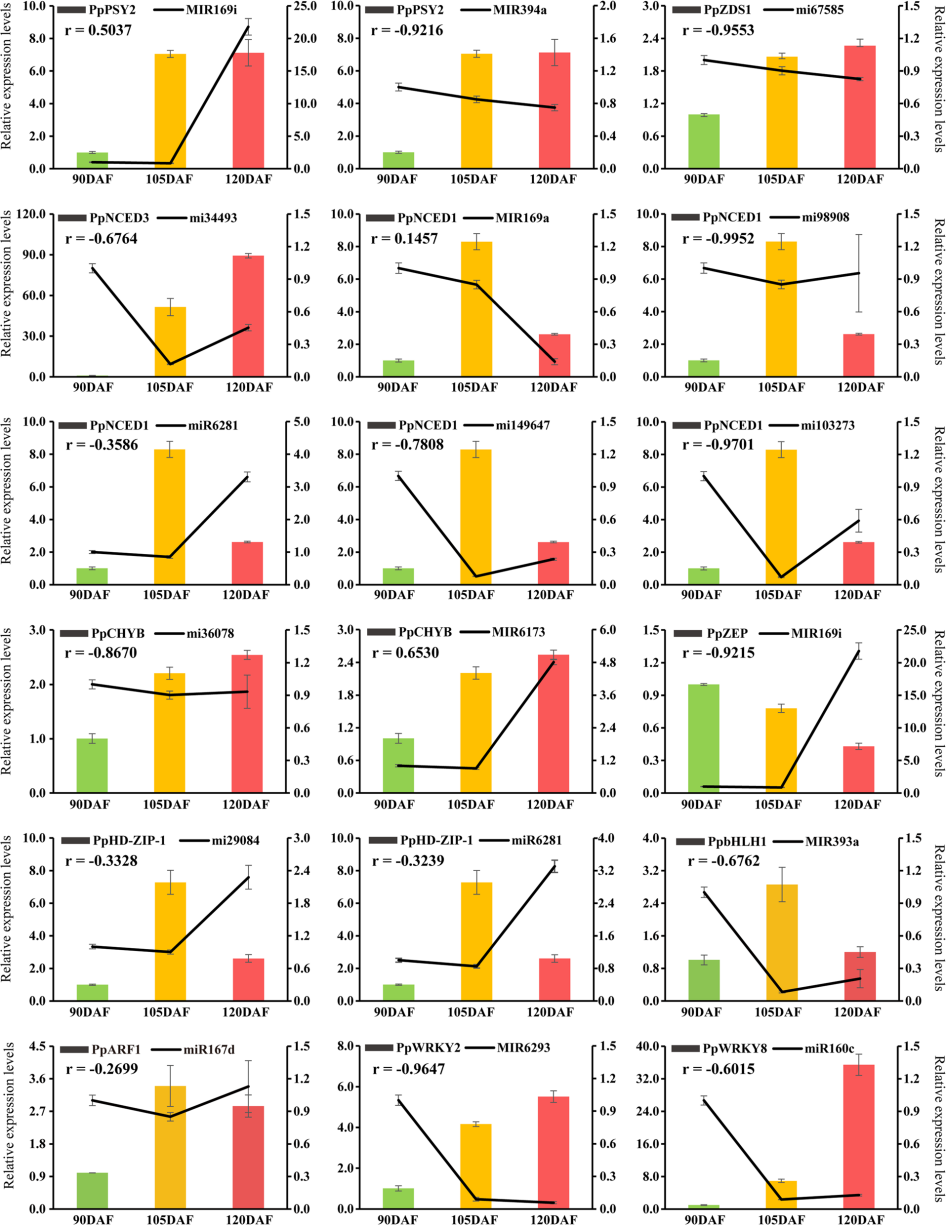
Quantitative Real-Time Polymerase Chain Reaction (qRT-PCR) analysis of miRNAs and target transcripts at different developmental stages. Detailed information on genes and miRNAs is available in Table S1

To gain deeper insights into the regulatory mechanisms governed by miRNAs in the carotenoid synthesis pathway, miRNAs and their target genes implicated in carotenoid synthesis were elucidated through miRNA sequencing and degradome sequencing. Coanalysis outcomes revealed a total of 14 miRNA‒mRNA pairs and 8 miRNA‒TF pairs as regulatory components within the carotenoid synthesis pathway of yellow peach peel. Among these, 11 miRNA‒mRNA pairs and 5 miRNA-TF pairs displayed an inverse expression pattern (Table S8). To corroborate these findings, qRT‒PCR analysis was conducted to validate the expression patterns of differentially expressed structural genes and TFs alongside miRNA regulatory components. The results indicated that MIR169i, MIR169a, and MIR6173 exhibited positive correlations with *PSY2*, *NCED1*, and *CHYB*, respectively. Conversely, the expression levels of the remaining 15 regulatory components exhibited a negative correlation with their respective target genes (Fig. 5). This observed pattern aligned closely with the expression profiles obtained from RNA-seq and miRNA-seq (Table S8), lending credence to the reliability of the sequencing data. It is noteworthy that the targets of these identified miRNAs consist of pivotal enzyme-encoding genes and prospective TFs integral to carotenoid synthesis. This suggests that miRNAs potentially orchestrate the participation of these target genes in the intricate process of carotenoid synthesis.

### The mdm-miR858 targets PpMYB9 to inhibit its activity

**Fig. 6:**
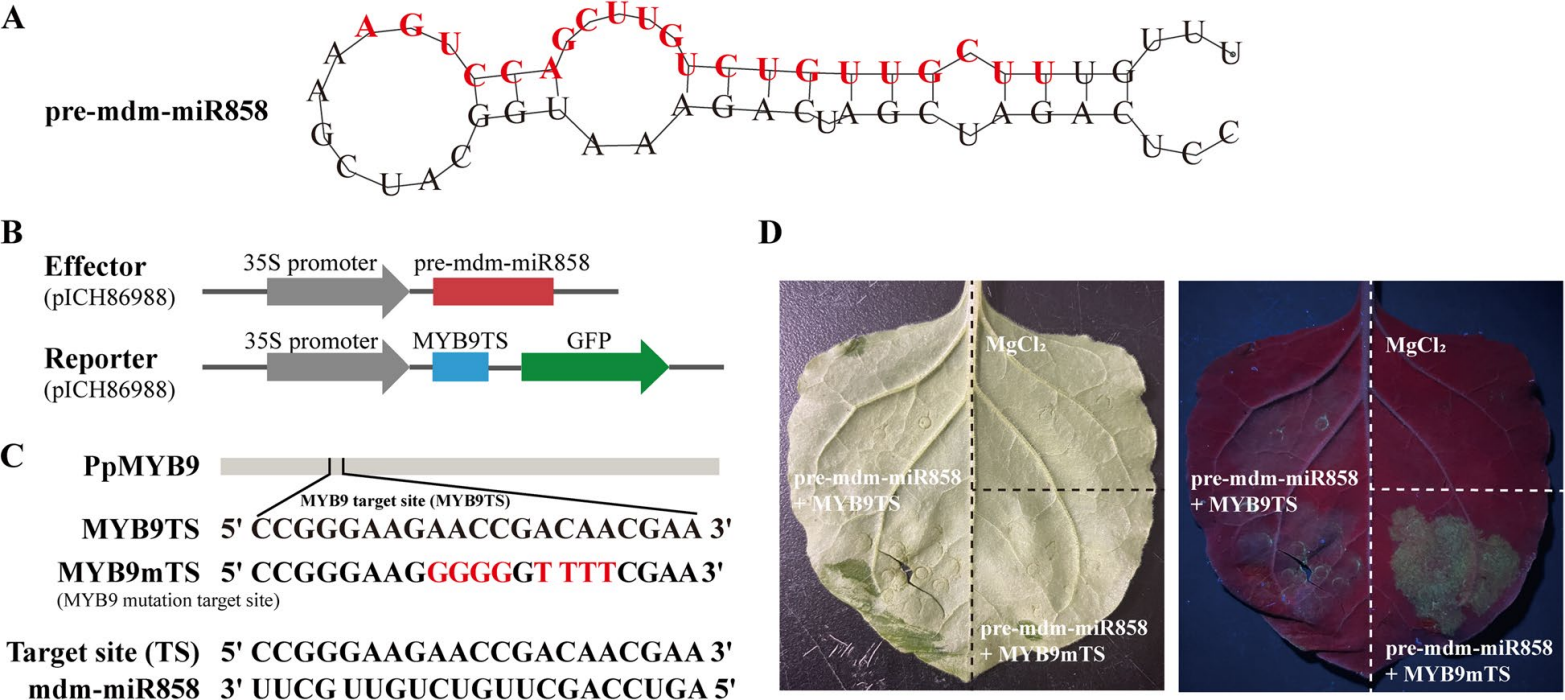
Transcripts of PpMYB9 are targeted by mdm-miR858. **A** RNA secondary structure analysis predicted the stem-loop structure formed by the mdm-miR858 precursor (pre-mdm-miR858). The mature mdm-miR858 sequence is shown in red. **B** The mdm-miR858 precursor sequence were inserted into the pICH86988 vector to form an effector, and the PpMYB9 target site (MYB9TS) and its mutant (MYB9mTS) fused with a GFP reporter gene were inserted into the pICH86988 vector to form a reporter plasmid. **C** Site analysis of mdm-miR858 targeting shear PpMYB9. MYB9mTS (negative control) is a mutant of MYB9TS. **D** Effect of mdm-miR858 on PpMYB9 activity. The coexpression of pre-mdm-miR858 with MYB9TS did not exhibit GFP signaling, whereas substitution of the MYB9mTS did not affect GFP expression

The RNA secondary structure analysis confirmed that the mdm-miR858 precursor could form a typical stem-loop structure (Fig. 6A, sequences of mature mdm-miR858 are indicated in red). The PpMYB9 coding sequence (CDS) contained a target-binding sequence that matches mature mdm-miR858 (Fig. 6C). The pre-mdm-miR858 (mdm-miR858 precursor sequence) sequences were inserted into the pICH86988 vector to form an activation plasmid, while the PpMYB9 target site (MYB9TS) and its mutant (MYB9mTS) were inserted into the pICH86988 vector fused with a GFP reporter gene to form a reporter plasmid (Fig. 6B). The results of the transient expression experiments showed that the coexpression of pre-mdm-miR858 with MYB9TS did not exhibit GFP signaling, whereas substitution of the MYB9mTS did not affect GFP expression (Fig. 6D). This result demonstrated that mdm-miR858 inhibits the expression of PpMYB9 by targeted cleavage. The primers are listed in Table S1.

## Discussion

### Carotenoid accumulation and color change in yellow peach peel

Fruit color represents a pivotal agronomic trait and significantly influences fruit quality. It serves as an indicator of fruit ripeness and stands as one of the key factors that captivate consumers and ascertain the market value of fruits (Jiang et al. 2019). Despite the abundance of carotenoids in peach flesh (Cao et al. 2017), studies investigating their variation and accumulation in the peel remain scarce. Throughout the ripening process of yellow peaches, the peel color typically undergoes a transition from green to yellow to orange‒red (Fig. 1A), with carotenoids playing a critical role in this transformation (Yuan et al. 2015). In this study, we meticulously analyzed the accumulation patterns of 14 carotenoids and 40 xanthophyll lipids in peach peel across three distinct developmental stages by employing UHPLC-APCI-MS/MS. Our investigation revealed the accumulation of diverse carotenoid types during each developmental stage (Fig. 1B), suggesting that the variations in these compounds could be intrinsic to the observed changes in peel color. Previous research has demonstrated that as pepper fruits mature, the degradation of flavonoids and chlorophyll within plastids lead to the gradual appearance of yellow or red hues attributed to accumulated carotenoids (Hugueney et al. 1996; Liu et al. 2020). This phenomenon potentially underlies the shift in peel color. In parallel, as yellow peach fruit matures, internal pigments experience degradation and assume the characteristic colors associated with carotenoids. A comparable phenomenon occurs in leaves, where the degradation of chlorophyll reveals potential colors arising from other pigments (Bliss et al. 2002). The analogous color transitions observed in yellow peach peel and these instances suggest that carotenoids play a significant role in determining peel color presentation.

### Differential expression of genes involved in carotenoid biosynthesis affects differential accumulation of carotenoids

Studies have indicated that the differential accumulation of carotenoids primarily stems from the distinct expression patterns of related enzyme-encoding genes (Galpaz et al. 2006; Liu et al. 2020). In this study, a collection of DEGs—namely, *PSY*, *ZDS*, *CRTISO*, *LCYB*, *LCYE*, *CHYB*, *VDE*, *ZEP*, and *NCED*—were pinpointed as key contributors to carotenoid biosynthesis within peach peel (Fig. 2A). Among these, PSY emerges as the foremost pivotal enzyme in carotenoid biosynthesis, significantly influencing overall carotenoid accumulation in plants (Huh et al. 2001). The heightened expression levels of *PSY2* that positively contributed to an increase in (E/Z)-phytoene content. This aligns with analogous findings in pepper, where Liu et al. (2020) delved into the transcriptome and metabolomics of four pepper cultivars, attributing the differential accumulation of phytoene content to the elevated expression of *PSY1*. Additionally, the levels of α-carotene, *β*-carotene, and γ-carotene were correlated with the expression levels of *LCYB* and *LCYE*. Of paramount significance, *β*-carotene and zeaxanthin, pivotal metabolites influencing fruit quality, are responsible for the distinctive yellow and orange hues seen in yellow peaches (Zhao et al. 2022). While the expression level of *LCYB* displayed an initial increase followed by a subsequent decrease with developmental stage, the *β*-carotene content did not mirror this trend. This suggests that *LCYB* might not be the direct cause of the decline in *β*-carotene content. Remarkably, the *β*-carotene content remained consistently across different developmental stages, potentially reflecting the irreplaceable role of *β*-carotene throughout the entire lifecycle of plants.

CHYB, a key enzyme catalyzing the conversion of *β*-carotene into zeaxanthin, demonstrated sustained expression, resulting in an augmented zeaxanthin content and thereby elevating the yellow color intensity of peach peel. A growing body of evidence underscores the active involvement of *CHYB* in carotenoid accumulation. Notably, in petals of yellow *Ipomoea* sp., higher *CHYB* expression and downstream product content were observed compared to white *I. obscura* and *I. nil* flowers (Yamamizo et al. 2010). A mutation in *CrtR-b2*, a *CHYB* homolog specifically expressed in tomato petals, yielded a white flower phenotype (Galpaz et al. 2006), further affirming the pivotal role of *CHYB* in carotenoid buildup. Moreover, the expression levels of *ZEP* and *VED* influenced the conversion between violaxanthin and zeaxanthin. Concomitantly, NCEDs orchestrated the cleavage of violaxanthin and neoxanthin to generate xanthoxin—the immediate C_15_ precursor of ABA (Cutler and Krochko 1999; Liotenberg et al. 1999). As peach fruit developed, the escalating expression levels of *NCED*s likely contributed to the gradual synthesis of neoxanthin into ABA. This hormone, ABA, assumes a vital role in diverse aspects of plant development, encompassing coloration, ripening, and the initiation of ethylene synthesis (Galpaz et al. 2008; Zhang et al. 2009; Sun et al. 2010).

### The new insights into the mechanism of carotenoid synthesis

WGCNA yielded novel insights into the regulatory network governing carotenoid synthesis in yellow peach peel. In tomato, *SGR* directly interacts with *SlPSY1* to oversee the accumulation of lycopene and *β*-carotene (Luo et al. 2013). A similar function might be attributed to the SGR gene identified in the pink module.

Beyond its role in imparting vibrant color, the peel establishes a homeostatic environment conducive to fruit development. Through WGCNA, *NIK1*, *ATL46*, *ZAT5*, *PDR1*, *PUB17*, and *BOI* emerged as potential key regulators in the response to environmental adversity (Table S5). In the context of *Arabidopsis*, *NIK1* operates in both plant development and defense (Santos et al. 2009) mechanisms. Similarly, *ShATL78L* in tomato has been recognized as an abiotic stress-responsive gene (Song et al. 2016). In apple, *MdZAT5* demonstrates dual effects—positively regulating anthocyanin accumulation while negatively impacting salt tolerance (Wang et al. 2022). PDR, belonging to the ABCG subfamily of ATP-binding cassette transporters, plays a role in safeguarding tobacco against fungal and bacterial pathogens (Bienert et al. 2012). SNI1, a negative regulator of systemic acquired resistance (Li et al. 1999), also contributes to the network. The E3 ubiquitin-protein ligase *BOI* elevates disease and abiotic stress resistance in *Arabidopsis* by ubiquitinating MYB (Luo et al. 2010). *In the context of* defense signaling in *Arabidopsis* (Yang et al. 2006), *PUB17* assumes significance, while *Lr10* is pivotal for rust resistance in wheat (Loutre et al. 2009).

Additionally, *PI-PLC6* and *PRODH2* may participate in growth and developmental processes of yellow peach peels. *PI-PLC6*, affiliated with the phosphatidylinositol-specific phospholipase C (PI-PLC) family, plays multifaceted roles encompassing pollen tube elongation (Dowd et al. 2006), hormone signaling, stress response, and pathogen defense in plants (Hunt et al. 2003; Vossen et al. 2010; Servet 2012; Li et al. 2017). PRODH2 extends its influence beyond proline catabolism, serving as a key player in energy provision, the translocation of redox potential between cellular compartments, and the generation of reactive oxygen species (Servet et al. 2012). These findings offer robust evidence and promising avenues for further research on the hub genes identified in this study. Furthermore, considering the chloroplast as the site of carotenoid synthesis and presence, genes such as SPPA, HMDS, FAF-Like, CAT6, STAY-GREEN, DHD/SHD, QORH, and ASA—which reside within the chloroplast—likely contribute to carotenoid anabolism (Table S5).

### TFs are involved in carotenoid synthesis in yellow peach peel

Several members within the MYB family have garnered attention due to their roles in carotenoid biosynthesis. In *Mimulus lewisii*, the R2R3-MYB TF "reduced carotenoid pigmentation 1" (*MlRCP1*) stands out as a key regulator of carotenoid biosynthesis genes within flowers (Sagawa et al. 2016). The regulation of carotenoid distribution in kiwifruit (*Actinidia deliciosa*) is orchestrated by *AdMYB7*, which activates *AdLCYB* expression (Ampomah-Dwamena et al. 2019). Orchids feature *Rhyncholaeliocattleya*-promoted carotenoid pigmentation 1 (*RcPCP1*), a factor intensifying *α*-carotene and lutein accumulation (Li et al. 2020a, b). Meanwhile, in *Camellia sinensis*, CsMYB110 has been identified as a carotenoid synthesis regulator. It clusters with MtWP1, AtPAP1, and AtMYB113, and its efficacy is authenticated through expression pattern and overexpression analysis (Meng et al. 2019; Li et al. 2022). Within this study, PpMYB1, PpMYB4, and PpMYB9 emerged as robust contenders for involvement in carotenoid synthesis, as substantiated by phylogenetic evolution and expression pattern analysis. The expression trends of PpMYB4 and PpMYB9 are inversely correlated with the accumulation of multiple carotenoids, mirroring the behavior of the homolog MtWP1. PpMYB1, clustering with CrMYB68, shares traits with its counterpart CrMYB68, which inhibits the conversion of *α*-branch and *β*-branch carotenoids in *Citrus reticulate* (Zhu et al. 2017). High PpMYB1 expression in this study aligns with decreased *α*- and *β*-branch carotenoid content (Fig. 2), suggesting a parallel function to CrMYB68. Moreover, the MBW complex also factors into carotenoid biosynthesis regulation (Meng et al. 2019). In tea, CsMYB110 mediates carotenoid biosynthesis through the MBW complex, and its overexpression increases the carotenoid content (Li et al. 2022).

Beyond the established role of MYB, other TFs, such as bHLH, NAC, WRKY, bZIP, and ARF, are also implicated in carotenoid synthesis. For instance, in citrus, CubHLH1 plays a role in carotenoid accumulation in fruits (Endo et al. 2016). Tomato SlNAC4 positively impacts carotenoid accumulation (Ma et al. 2014; Zhu et al. 2014). *Osmanthus fragrans*, OfWRKY3 positively regulates *OfCCD4*, a carotenoid cleavage dioxygenase gene, governing carotenoid catabolism (Han et al. 2016). SlWRKY35 augments carotenoid accumulation by activating *SlDXS1* during tomato fruit ripening (Yuan et al. 2022). The role of HY5 regulating *PSY* transcription is facilitated through direct binding to the G-box region of the promoter (Toledo-Ortiz et al. 2014). CmWRKY49, governing *CmPSY1*, fosters *β*-carotene accumulation in orange-fleshed oriental melon (Duan et al. 2022). IbARF5 influences carotenoid biosynthesis in *Ipomoea batatas* (Kang et al. 2018). Additionally, CsHB5 enhances ABA biosynthesis by activating *NCED2* transcription in citrus (Zhang et al. 2021). Collectively, these studies provide compelling evidence for the roles of bHLH62, NAC83, WRKY53, WRKY45, bZIP11, ARF6, and even LBD TFs in carotenoid synthesis within yellow peach peel. The potential involvement of LBD TFs in several developmental processes in plants underscores their plausible role in carotenoid synthesis (Han et al. 2021; Jia et al. 2022; Liang et al. 2022; Teng et al. 2022; Tian et al. 2022). These findings cement these TFs as promising candidates for delving into carotenoid synthesis mechanisms.

### MiRNAs are regulators of carotenoid synthesis in yellow peach peel

**Fig. 7:**
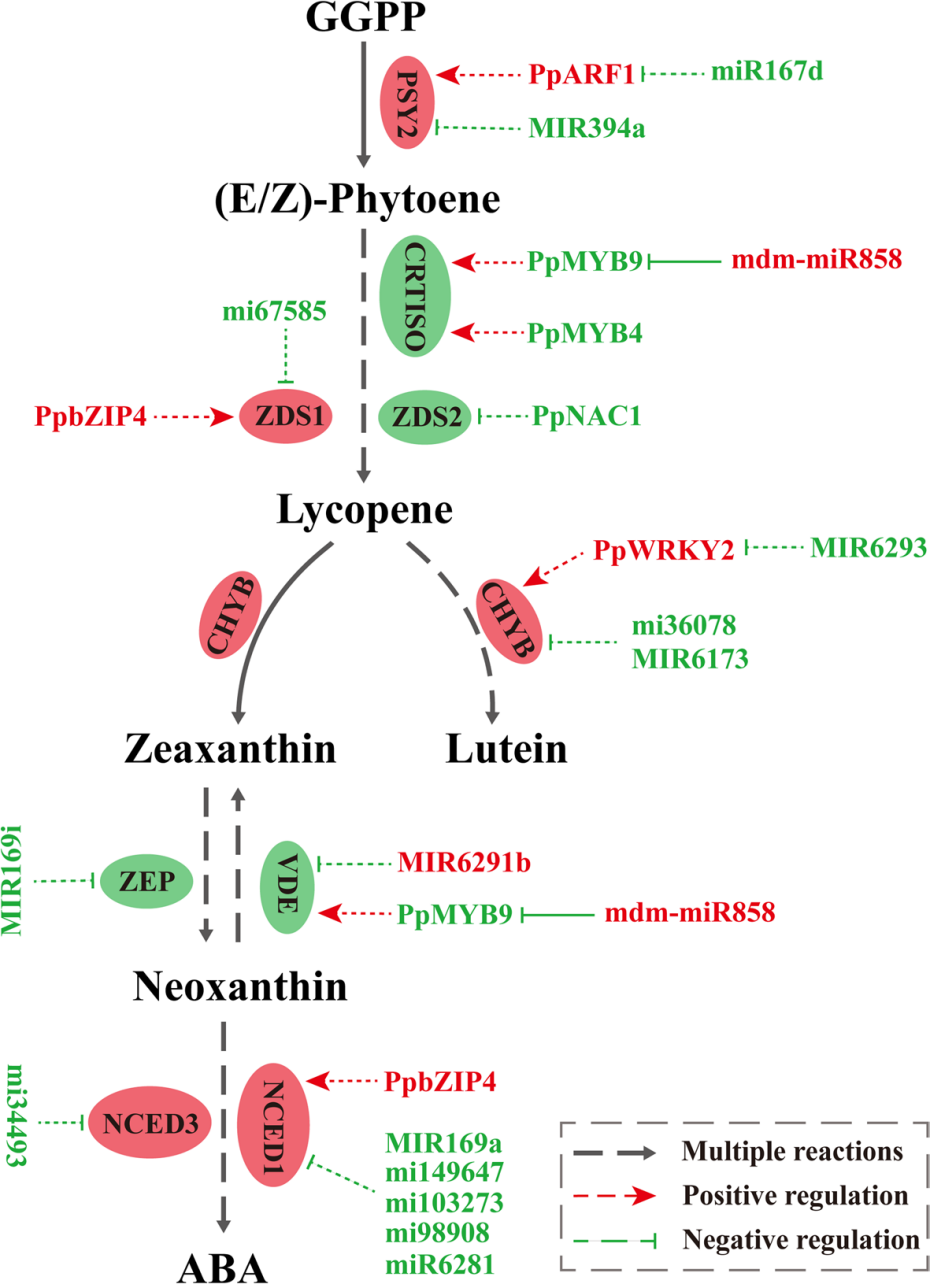
miRNA-mediated carotenoid synthesis in yellow peach peel. This regulatory network portrays how miRNAs influence the expression of enzyme-encoding genes and carotenoid accumulation via mediation of transcription factors (TFs) and structural genes

MiRNAs typically govern gene expression by either cleaving target mRNAs or suppressing target gene expression (Wu 2013). MiRNA sequencing predicted target genes of miRNAs, and the miRNA‒mRNA targeting association was validated through degradome analysis. Prior research has revealed that various miRNAs target TFs pivotal in plant development, such as miR156 for SBP, miR164 for NAC, and miR394 for F-box genes (Xu et al. 2010). MiRNAs influence carotenoid biosynthesis either by directly targeting or indirectly modulating the expression of structural genes and TFs (Gao et al. 2015; Koul et al. 2016). In our study, mdm-miR858 suppressed PpMYB9 gene expression by targeted cleavage (Fig. 6D). Our findings shed light on the potential roles of miRNAs in carotenoid synthesis. The shifts in carotenoid content correspond to the varying expression of structural genes and TFs under the control of specific miRNAs. For instance, miR167d modulates *PSY2* expression by mediating PpARF1. PpbZIP4, targeted by MIR11113, positively influences *NCED1* expression, bolstering ABA accumulation during fruit ripening. Similarly, miR2105 intervenes via OsbZIP86 to regulate *NCED3* expression, thereby affecting ABA content in rice (Premachandran 2022). By integrating transcriptome, miRNA-seq, degradome, and gene expression data, we propose a regulatory network encompassing miRNAs and their targets (Fig. 7), thereby unveiling the regulatory interactions in carotenoid biosynthesis within yellow peach peel. Key enzyme-encoding genes within the carotenoid synthesis pathway, including *PSY2*, *CRTISO*, *ZDS1/2*, *CHYB*, *ZEP*, *VDE*, and *NCED1/3*, are under the influence of one or more miRNAs and TFs in this network. Considering the valuable nutritional significance of carotenoids and their role in fruit coloration, elucidating the molecular mechanisms of miRNA involvement in carotenoid biosynthesis is poised to become a prominent avenue of research. Our study results offer substantial insight into miRNA involvement in carotenoid synthesis. However, validating these targets necessitates an effective gene transformation system for manipulating miRNA and TF expression in peaches, an aspect currently absent.

In summary, yellow peaches stand as exceptional sources of dietary carotenoids, including their peel, boasting abundant content. This study thoroughly and systematically assessed the carotenoid profiles and concentrations in yellow peach peel, concurrently constructing a plausible regulatory network of miRNAs and TFs orchestrating carotenoid synthesis. The network includes miR167d, MIR394a, mi67585, and mdm-miR858, governing carotenoid synthesis by targeting genes such as *PSY2*, *ZDS1/2*, *CHYB*, *ZEP*, *VDE*, and *NCED1/3*, along with TFs PpARF1, PpNAC1, and PpWRKY2. Ultimately, our study elucidates the molecular regulatory framework of carotenoid synthesis in yellow peach peel, furnishing a theoretical foundation and target genes for future genetic engineering endeavors aimed at cultivating high-carotenoid-content yellow peaches.

## Methods

### Sampling of plant material and RNA extraction

Fruit samples of yellow peach (*Prunus persica* L. cv. Huangjinmi) were harvested at 90DAF, 105DAF, and 120DAF days after bloom. Nine peach trees exhibiting consistent growth and fruit production were randomly allocated into three groups, resulting in three replicates. Subsequently, 27 fruits were randomly collected from each group, following a randomized group design. The peel, with a thickness of 0.5 mm, was carefully extracted from the fruit surface and immediately frozen in liquid nitrogen, thereafter stored at -80 °C.

Total RNA was extracted and purified using TRIzol Reagent (Invitrogen, Carlsbad, USA). To eliminate genomic DNA contamination, all RNA samples were treated with DNase I (TaKaRa, Dalian, China). Evaluation of RNA quality and purity was conducted through 1.0% (p/v) agarose gel electrophoresis and a NanoDrop 2000 spectrophotometer (Thermo, USA). Transcriptome and miRNA-seq analyses were conducted using total RNA from each sample. Total RNA sourced from different samples was pooled for degradome sequencing.

### Determination of carotenoid composition

Carotenoid composition analysis was detected by MetWare (Wuhan, China). Cryopreserved peach pericarp samples were pulverized with liquid nitrogen and subsequently freeze-dried. Next, then 50 mg of the dried powder was subjected to extraction by adding a mixture of hexane, acetone, and ethanol in a 1:1:2 (v/v/v) ratio along with an internal standard. The extraction solution was vigorously mixed for 20 min at room temperature, followed by collection of the supernatant. After being dried using a nitrogen blowing instrument, the supernatant was reconstituted using a mixture of methanol and methyl tert-butyl ether in a 3:1, (v/v) ratio. The resultant solution was then subjected to filtration through a 0.22 μm membrane and subsequently analyzed by LC-APCI-MS/MS system.

For the analysis, the extracted sample was again mixed for 20 min at room temperature, followed by centrifugation to collect the supernatant. This supernatant was then dried using a nitrogen blowing instrument and reconstituted with a mixture of methanol and methyl tert-butyl ether in a 3:1 (v/v) ratio. The resulting solution was filtered through a 0.22 μm membrane and subjected to analysis using the LC-APCI-MS/MS system (UHPLC, ExionLC AD, https://sciex.com.cn/; MS. Applied Biosystems 6500 Triple Quadrupole, https://sciex.com.cn/). HPLC Conditions: the chromatographic column utilized was YMC C30 (3 µm, 2 mm × 100 mm). Solvent system was methanol: acetonitrile (1:3, v/v) with 0.01% BHT and 0.1% formic acid (A), methyl tert-butyl ether with 0.01% BHT (B). Gradient program: started at 0% B (0–3 min), increased to 70% B (3–5 min), then increased to 95% B (5–9 min), finally ramped back to 0% B (11–12 min). The analysis was carried out at a temperature of 28 °C with a flow rate of 0.8 mL/min, and the injection volume was set at 2 µl. MS analysis was performed using the API 6500 Q TRAP LC‒MS/MS System, equipped with an APCI Turbo Ion-Spray interface, operating in a positive ion mode and controlled by Analyst 1.6.3 software (AB Sciex). The APCI source operation parameters were as follow: source temperature 350 °C, curtain gas (CUR) were set at 25.0 psi.

### Transcriptome sequencing

For transcriptome analysis, sequencing libraries were prepared following Illumina's kit protocols. Initially, poly(A) mRNA was isolated from total RNA utilizing oligo(dT) magnetic beads. Subsequently, the mRNA was fragmented using the RNA Fragmentation Kit. The first strand of cDNA was synthesized using a combination of six random hexamer primers and short fragment templates. The second cDNA strand was then generated employing a buffer, dNTPs, RNase H, and DNA polymerase I. This double-stranded cDNA was ligated to sequencing adapters. Afterwards, appropriately sized cDNA fragments were isolated through gel electrophoresis analysis, followed by PCR enrichment. The resulting product was loaded onto the Illumina HiSeq4000 platform for transcriptome sequencing. To enhance data quality, reads with low quality and adapter sequences were trimmed using the Illumina Pipeline. Functional annotation of transcripts was carried out by comparing the sequenced reads to the *Prunus persica* genome.

### Transcription factor screening and phylogenetic tree

Correlation analysis was conducted using the expression levels of all TFs in relation to carotenoid content. TFs with an absolute |PCC| exceeding 0.9 were selected, taking into account both positive and negative regulatory relationships of TFs. These identified TFs were further examined for their correlation with differentially expressed structural genes associated with carotenoid synthesis. Among these, TFs exhibiting |PCC| values greater than 0.9 were shortlisted as potential candidate TFs. The coding sequence of all genes were extracted from the transcriptome data. From these sequences, the longest open reading frames were extracted and translated into protein sequences for the subsequent construction of evolutionary trees. The neighbor-joining phylogenetic tree was constructed using MEGA X software, and the process involved performing 1000 bootstrap replications to enhance the statistical robustness of the tree topology.

### Coexpression network analysis for construction of modules

To identify genes associated with carotenoid synthesis, carotenoids that exhibited differential accumulation across three developmental stages were chosen as the basis for WGCNA alongside all differentially expressed genes (|PCC|> 0.9) in accordance with default parameters. The WGCNA was carried out utilizing the BMKCloud Platform. The resulting coexpression network was visualized using Cytoscape software.

### miRNA sequencing, degradome sequencing and coanalysis

MiRNA-seq and degradome-seq analyses were conducted by Lianchuan Bio (Hangzhou, China). For miRNA-seq, nine sRNA sequencing libraries were prepared using TruSeq Small RNA Sample Prep Kits (Illumina, San Diego, USA) and sequenced on the Illumina Hiseq2000/2500 platform with a read length of 1 X 50 bp. Raw reads underwent processing with an in-house program, ACGT101-miR (LC Sciences, Houston, Texas, USA), to eliminate adapter dimers, junk, low-complexity sequences, common RNA families (rRNA, tRNA, snRNA, snoRNA), and repeats. Following this, unique sequences of 18–25 nucleotide in length were aligned to specific species precursors in miRBase 22.0 using a BLAST search. This alignment aimed to identify both known miRNAs and novel 3p- and 5p-derived miRNAs. Mismatches within the sequence and variations in length at both the 3’ and 5’ ends were permitted during alignment. Sequences uniquely mapping to mature miRNAs in the hairpin arms were categorized as known miRNAs, whereas sequences mapping to the opposite arm of the annotated mature miRNA-containing arm were considered novel 5p- or 3p-derived miRNA candidates. The remaining sequences were aligned to selected species precursors (excluding the specific species) in miRBase 22.0 using a BLAST search, and the mapped pre-miRNAs were further BLASTed against the genomes of specific species to identify their genomic locations. Expression levels of miRNAs were quantified using transcripts per kilobase of exon model per million mapped reads (TPM). Differential expression miRNAs were defined as those with a false discovery rate (FDR) < 0.01 and |log2 (FC, fold change) |≥ 1.

For degradome-seq, nine total RNA samples were equally pooled for the construction of degradome sequencing libraries. Poly(A) RNA was isolated and annealed with biotinylated random primers. The annealed products containing 5’-monophosphates were ligated to a 5’-adaptor, and first-strand cDNA was generated. Single-end sequencing (36 bp) using the 5’-adapter only was performed on an Illumina Hiseq2500 at LC-BIO (Hangzhou, China). Data analysis was carried out using CleaveLand 4.0.

### Transient coexpression assay

The precursor stem-loop sequence of mdm-mi858 (pre-mdm-miR858) was inserted into the pICH86988 plasmid containing the CaMV 35S promoter by Golden Gate cloning. The PpMYB9 target site (MYB9TS) was co-inserted with GFP between the BsaI sites in pICH86988 to form a fusion protein. Agrobacterium tumefaciens containing pre-mdm-miR858 and MYB9TS was resuspended in suspension (10 mM MES, 10 mM MgCl2, and 150 μM acetosyringone), mixed, and then injected into *N. benthamiana* leaves. The infested tobacco was incubated at 24 °C under 16-h light/8-h darkness, and after 3 days, the fluorescence was observed after irradiation using a hand-held excitation light source (LUYOR-3280, LUYOR). The mutant sequence MYB9mTS was used as a negative control. To correctly insert MYB9TS and MYB9mTS into the plasmid, AATG and CGAA sites were added at the ends of the forward and reverse primers of the target sequences, respectively. The primers are listed in Table S1.

### qRT‒PCR analysis

To assess the reliability of miRNA‒mRNA target pairs, qRT‒PCR analysis was conducted on both DEGs and miRNAs following established procedures (Zheng et al. 2022). Three independent biological triplicates, each comprising three technical replicates, were performed. The LineGene 9600 Plus Fluorescent Quantitative PCR System (BIOER, Hangzhou, China) was utilized for perform all qRT‒PCR experiments. Total RNA extraction from each sample was carried out using the RNAprep Pure Plant Plus Kit (Tiangen, Beijing, China). Primers designed using the Integrated DNA Technologies tool (https://sg.idtdna.com/site/Order/oligoentry/set) for qRT‒PCR are listed in Table S1. As per the manufacturer's guidelines, cDNA was synthesized using the HiScript II One Step RT‒PCR Kit (Vazyme, Nanjing, China) and then diluted 10 times for use as a qRT‒PCR template. For miRNA quantification, total RNA was reverse transcribed into cDNA using the Mir-X miRNA First-Strand Synthesis Kit (Takara, Beijing, China). The MicroRNAs qPCR Kit (Sangon Biotech, Shanghai, China) was employed for all miRNA qPCR experiments, with U6 serving as the internal control. In mRNA qPCR experiments, SYBR Green PCR Master Mix (Vazyme, Nanjing, China) was used, and the *PpTEF2* gene was employed as the internal control (Tong et al. 2009). The 2^–ΔΔCt^ method was employed to calculate the relative expression changes of mature miRNAs and genes. Student's t-test was applied to assess the statistical differences between qRT-PCR results from two samples (*P* < 0.05).

### Supplementary Information

**Additional file 1: Table S1.** Primers employed in this study.

**Additional file 2: Table S2.** Content of all carotenoids and DACs.

**Additional file 3: Table S3.** The DEGs involved in carotenoids synthesis of 90DAF_vs_105DAF, 90DAF_vs_120DAF and 105DAF_vs_120DAF.

**Additional file 4: Table S4.** Lists of DEGs with DACs PCC ≥ 0.90 and ≤ -0.90.

**Additional file 5: Table S5.** Comprehensive information of all genes within salmon, pink and cyan modules.

**Additional file 6: Table S6.** List of genes in the correlation network of salmon, pink and cyan modules.

**Additional file 7: Table S7.** TFs associated with carotenoids and DEGs involved in carotenoid synthesis.

**Additional file 8: Table S8.** Identification of miRNA‒target genes pairs (TFs and structure genes) through degradome analysis in yellow peach peel.

**Additional file 9: Fig. S1.** Accumulation patterns of all carotenoid metabolites in yellow peach peel.

**Additional file 10: Fig. S2.** Expression patterns of key hub genes and transcription factors (D) within pink (A), cyan (B) and salmon (C) modules.

**Additional file 11: Fig. S3.** Phylogenetic tree (A) and expression patterns (B) of predicted MYB TFs involved in carotenoid biosynthesis.

**Additional file 12: Fig. S4.** Identification of *cis*-elements in promoters of carotenoid biosynthesis genes. The promoter sequences were isolated from peach genome (https://genome.jgi.doe.gov/portal/pages/dynamicOrganismDownload.jsf?organism=Ppersica), and the *cis*-elements analysis were performed in New PLACE (https://www.dna.affrc.go.jp/PLACE/) and PlantCare (https://bioinformatics.psb.ugent.be/webtools/plantcare/html/).

## Data Availability

All data supporting the findings of this study are included in the manuscript and its supplementary information.

## Abbreviations

TFs: Transcription factors
qRT-PCR: Quantitative real-time PCR
WGCNA: Weighted gene co-expression network analysis
DACs: Differentially accumulated carotenoids
DEGs: Differentially expression genes
GGPP: Geranylgeranyl diphosphates
PSY: Phytoene synthase
PDS: Phytoene desaturase
Z-ISO: *ζ*-Carotene isomerase
ZDS: *ζ*-Carotene desaturase
CRTISO: Carotenoid isomerase
LCYE: Lycopene *ε*-cyclase
LCYB: Lycopene *β*-cyclase
CHYB: *β*-Carotene hydroxylase
CYP97A: Cytochrome P450-type monooxygenase 97A
ZEP: Zeaxanthin epoxidase
VDE: Violaxanthin de-epoxidase
NXS: Neoxanthin synthase
NCED: 9-Cis-epoxycarotenoid dioxygenase
