# Supplementary figures and images for: Multiomics analysis provides new insights into the regulatory mechanism of carotenoid biosynthesis in yellow peach peel
Source: Mol Hortic. 2023 Nov 3;3:23. doi: 10.1186/s43897-023-00070-3 (PMC10623742; doi:10.1186/s43897-023-00070-3)

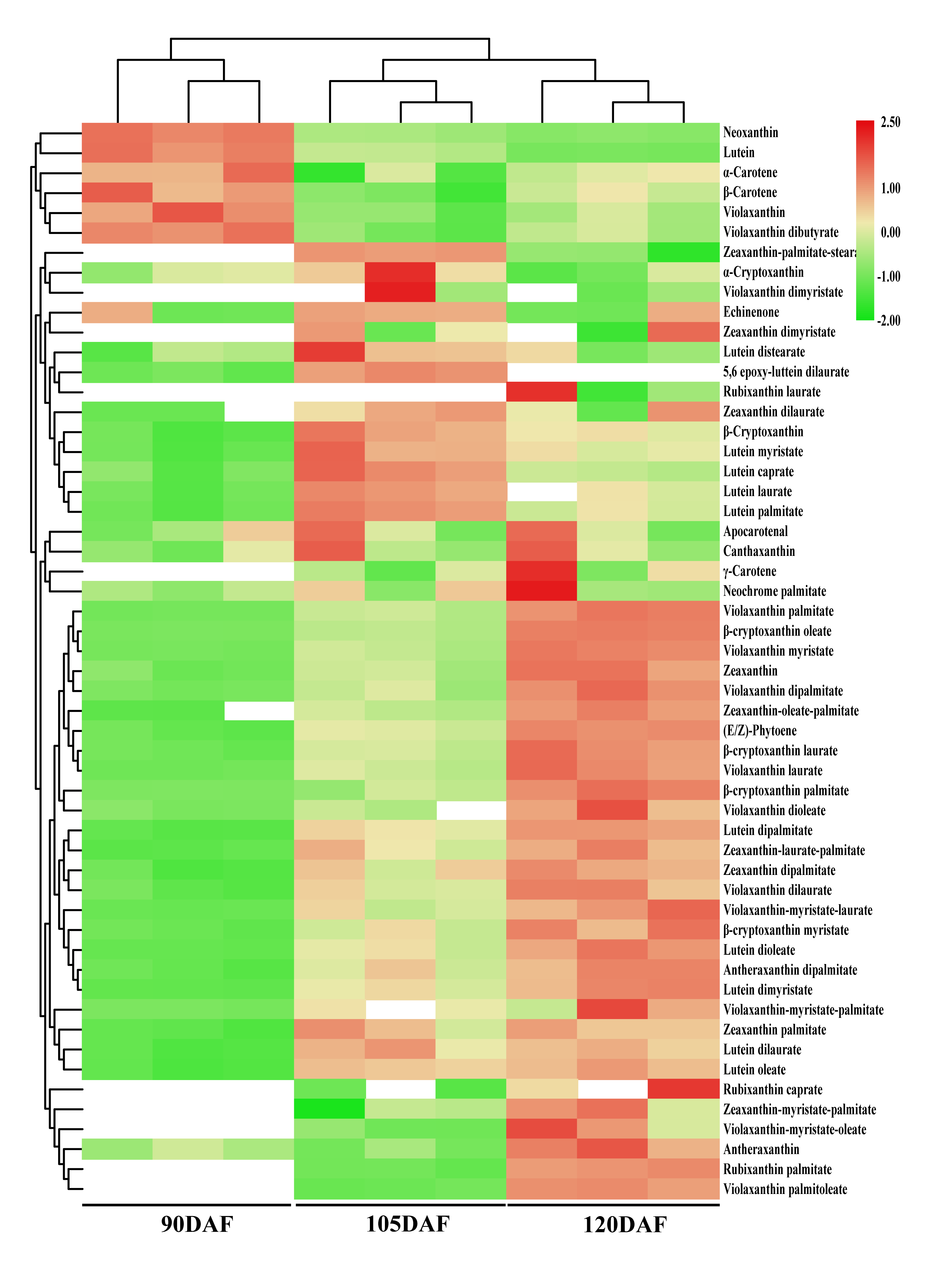

Supplement: Supplementary file 9 — Additional file 9: Fig. S1. Accumulation patterns of all carotenoid metabolites in yellow peach peel. [file 43897_2023_70_MOESM9_ESM.tif]

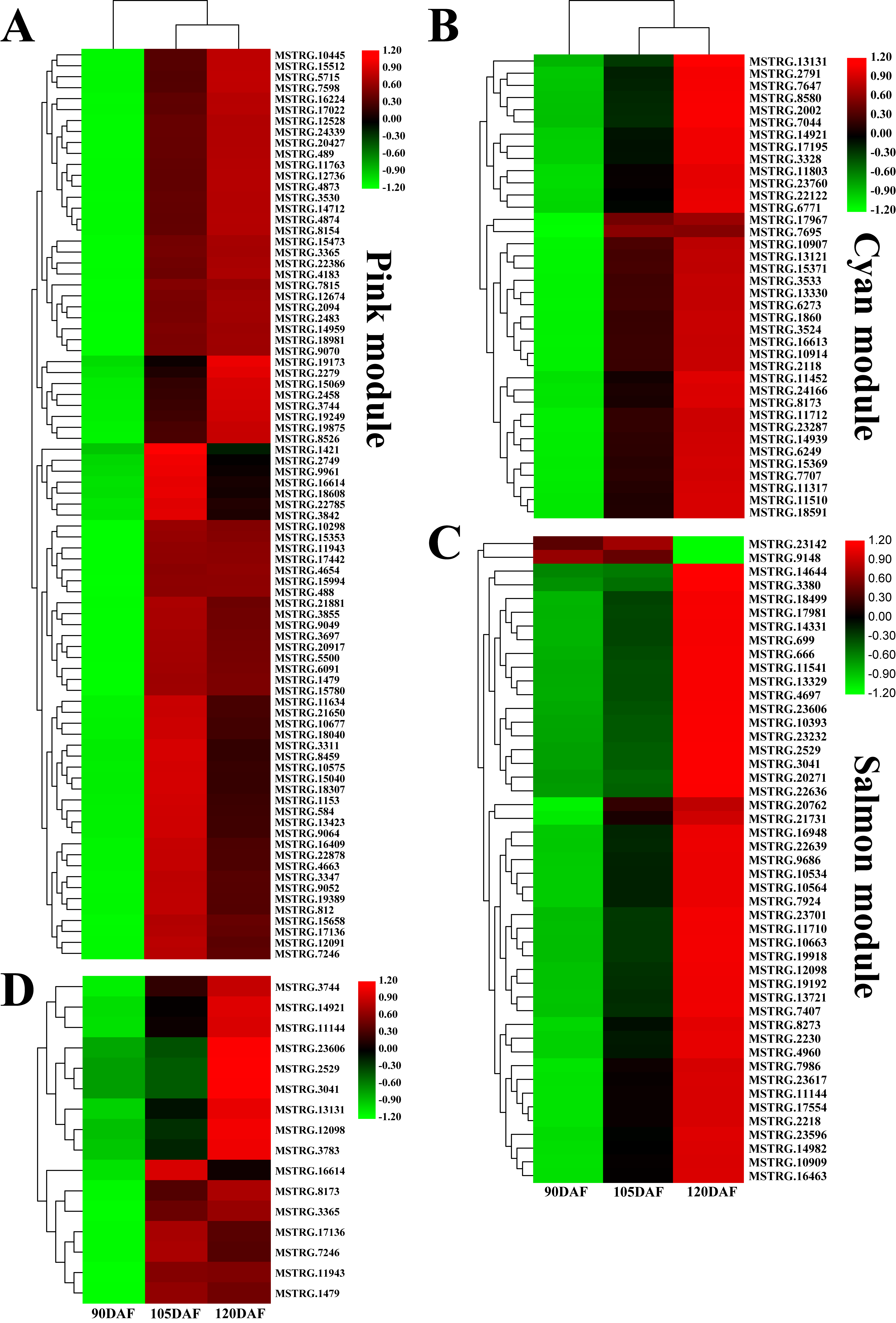

Supplement: Supplementary file 10 — Additional file 10: Fig. S2. Expression patterns of key hub genes and transcription factors (D) within pink (A), cyan (B) and salmon (C) modules. [file 43897_2023_70_MOESM10_ESM.tif]

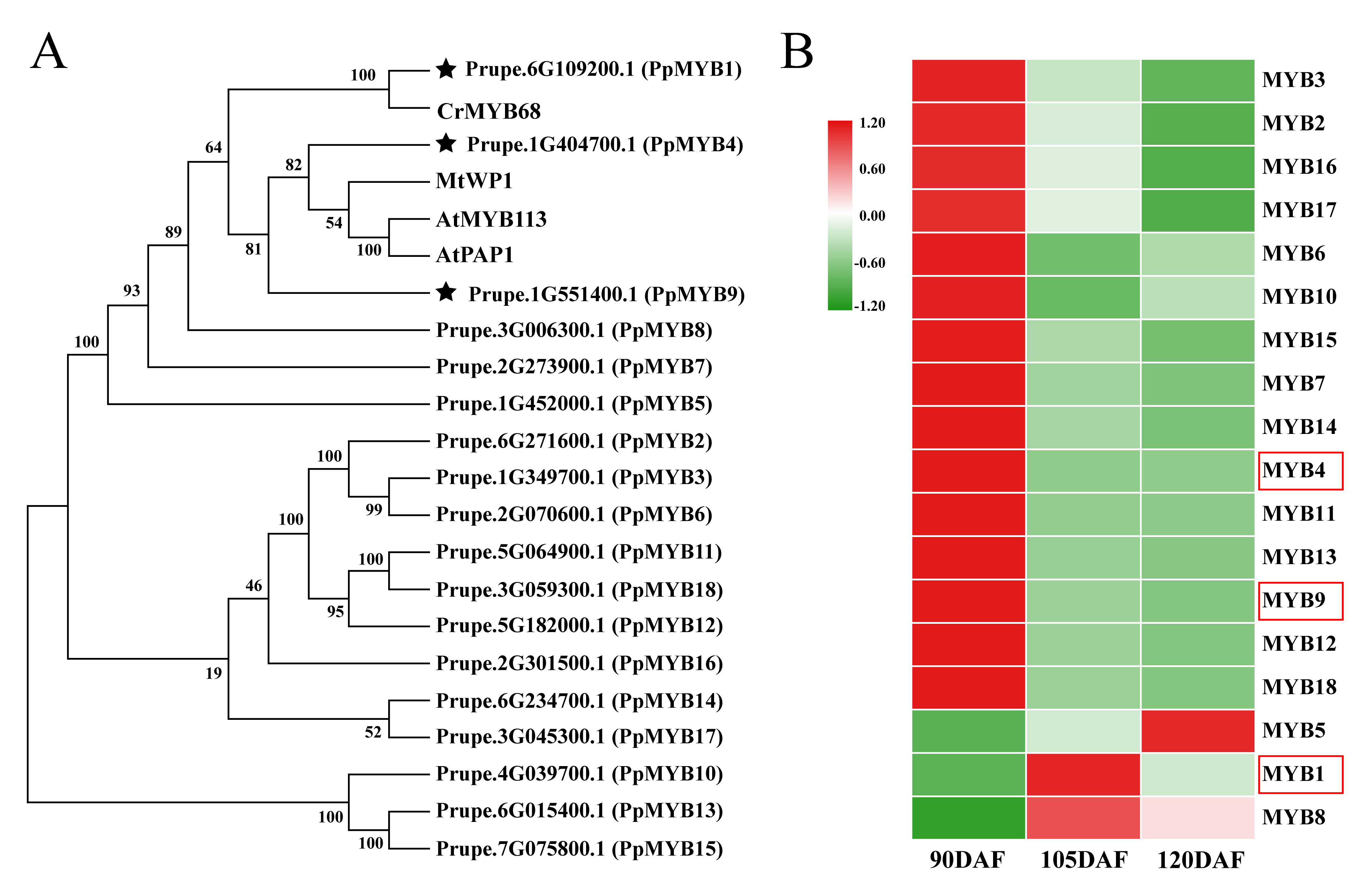

Supplement: Supplementary file 11 — Additional file 11: Fig. S3. Phylogenetic tree (A) and expression patterns (B) of predicted MYB TFs involved in carotenoid biosynthesis. [file 43897_2023_70_MOESM11_ESM.tif]

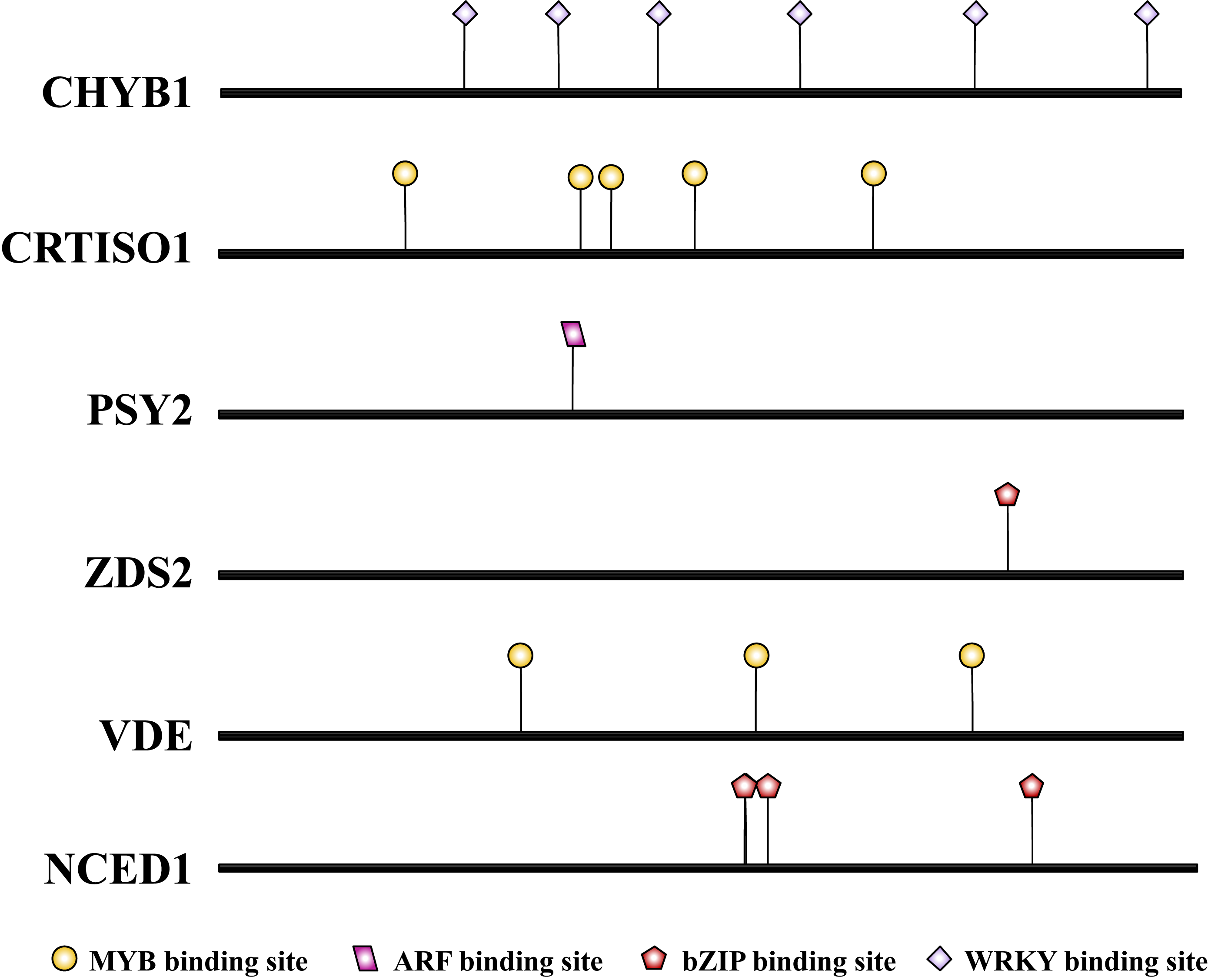

Supplement: Supplementary file 12 — Additional file 12: Fig. S4. Identification of cis-elements in promoters of carotenoid biosynthesis genes. The promoter sequences were isolated from peach genome (https://genome.jgi.doe.gov/portal/pages/dynamicOrganismDownload.jsf?organism=Ppersica), and the cis-elements analysis were performed in New PLACE (https://www.dna.affrc.go.jp/PLACE/) and PlantCare (https://bioinformatics.psb.ugent.be/webtools/plantcare/html/). [file 43897_2023_70_MOESM12_ESM.tif]
